# Fast Periodic Visual Stimulation EEG Reveals Reduced Neural Sensitivity to Fearful Faces in Children with Autism

**DOI:** 10.1007/s10803-019-04172-0

**Published:** 2019-08-29

**Authors:** Stephanie Van der Donck, Milena Dzhelyova, Sofie Vettori, Hella Thielen, Jean Steyaert, Bruno Rossion, Bart Boets

**Affiliations:** 1 Center for Developmental Psychiatry, Department of Neurosciences, KU Leuven, Leuven, Belgium; 2 Leuven Autism Research (LAuRes), KU Leuven, Leuven, Belgium; 3 Institute of Research in Psychological Sciences, Institute of Neuroscience, Université de Louvain, Louvain-La-Neuve, Belgium; 4 Department of Brain and Cognition, KU Leuven, Leuven, Belgium; 5 Université de Lorraine, CNRS, CRAN, Nancy, France; 6 Université de Lorraine, CHRU-Nancy, Service de Neurologie, Nancy, France

**Keywords:** Autism, EEG, Face inversion effect, Facial emotion processing, FPVS, Implicit fear detection

## Abstract

We objectively quantified the neural sensitivity of school-aged boys with and without autism spectrum disorder (ASD) to detect briefly presented fearful expressions by combining fast periodic visual stimulation with frequency-tagging electroencephalography. Images of neutral faces were presented at 6 Hz, periodically interleaved with fearful expressions at 1.2 Hz oddball rate. While both groups equally display the face inversion effect and mainly rely on information from the mouth to detect fearful expressions, boys with ASD generally show reduced neural responses to rapid changes in expression. At an individual level, fear discrimination responses predict clinical status with an 83% accuracy. This implicit and straightforward approach identifies subtle deficits that remain concealed in behavioral tasks, thereby opening new perspectives for clinical diagnosis.

**Electronic supplementary material:**

The online version of this article (10.1007/s10803-019-04172-0) contains supplementary material, which is available to authorized users.

## Introduction

Social behavior and communication are largely determined by the efficient use and interpretation of nonverbal cues (Argyle 1972), such as facial expressions. Emotional face processing has often been studied in individuals with autism spectrum disorder (ASD), a neurodevelopmental disorder characterized by impaired reciprocal social communication and interaction, including deficient non-verbal communicative behavior (American Psychiatric Association 2014).

## Facial Emotion Processing Strategies in ASD

An abundance of behavioral studies has investigated emotion recognition in individuals with and without ASD, yielding mixed results in terms of group differences (Harms et al. 2010; Lozier et al. 2014; Uljarevic and Hamilton 2013). Deficits in fear recognition, for instance, have often been shown in adults with ASD (Humphreys et al. 2007; Pelphrey et al. 2002; Rump et al. 2009; Wallace et al. 2008), whereas child studies often reported intact fear processing in ASD (Evers et al. 2015; Lacroix et al. 2014; Law Smith et al. 2010; Tracy et al. 2011). Due to the ongoing development of fear recognition abilities during childhood, floor effects in both ASD and control children might conceal possible group differences until they emerge during adulthood.

The use of alternative, less automatic processing strategies in ASD (Harms et al. 2010) might affect expression recognition. Perceptual processing styles are commonly investigated using the face-inversion paradigm, as inversion of the face disrupts the typical holistic or configural face processing (Rossion 2008; Tanaka and Simonyi 2016). Reports of an absent face inversion effect in ASD (Behrmann et al. 2006; Gross 2008; Rosset et al. 2008) suggest the use of an atypical, more local and feature-based (emotion) processing style. However, other studies reported better emotion recognition in upright versus inverted faces, both in ASD and TD participants (McMahon et al. 2016; Wallace et al. 2008), indicating that participants with ASD are capable of holistic or configural face processing.

Difficulties in emotion processing may also occur when one fails to inspect the most relevant facial cues (Ellison an Massaro 1997). The eyes have been suggested to play a crucial role in fear recognition (Bombari et al. 2013; Wegrzyn et al. 2017), but also the importance of the mouth, and the combination of both these regions, has been emphasized (Beaudry et al. 2014; Eisenbarth and Alpers 2011; Gagnon et al. 2014; Guarnera et al. 2015). Results on the most informative facial features for emotion processing in ASD versus TD are inconclusive. Some studies demonstrated reliance on different facial cues for emotion recognition (Grossman and Tager-Flusberg 2008; Neumann et al. 2006; Spezio et al. 2007), whereas other studies showed that both groups employ the same facial information (Leung et al. 2013; McMahon et al. 2016; Sawyer et al. 2012). Still, a similar way of looking at faces for reading emotions does not automatically imply similar neural processing, nor a similar level of emotion recognition performance (Sawyer et al. 2012).

## Event Related Potential studies

To understand the neural basis of facial emotion processing in ASD, many researchers have measured Event-Related Potentials (ERPs) using electroencephalography (EEG) (Jeste and Nelson 2009; Luckhardt et al. 2014), but generally fail to draw consistent conclusions (Black et al. 2017; Monteiro et al. 2017).

One ERP component of particular interest for (expressive) face processing is the N170 (Hinojosa et al. 2015). Kang et al. (2018) proposed this ERP component as a possible neural biomarker of the face processing impairments in individuals with ASD. However, the differences in N170 found between ASD and TD groups could merely reflect a slower general processing of social stimuli (Vettori et al. 2018) or they could be caused by carryover effects from changes in the immediately preceding P100 component (Hileman et al. 2011). In addition, atypicalities in the N170 response to emotional faces may not be autism-specific: similar atypicalities have been observed in other psychiatric and neurological disorders and may rather be an indication of emotional face processing dysfunction as a symptom of these diagnoses, than disorder-specific deficits (Feuerriegel et al. 2015).

The use of visual mismatch negativity (vMMN) paradigms has also been suggested as a clinically relevant application (Kremláček et al. 2016). However, the low number of oddballs and the low signal-to-noise ratio (SNR) of classic ERP measurements require many trials, resulting in long EEG recordings. Furthermore, to be valuable and reliable as a clinical tool, measurements should be consistent across studies and participants, in order to facilitate individual assessment. Yet, the variable expression of the vMMN in terms of individual timing and format (Kremlácek et al. 2016) hampers the objective marking of the vMMN, especially at an individual level.

## Fast Periodic Visual Stimulation EEG

To overcome these difficulties, we used a relatively novel approach in the emotion-processing field, combining fast periodic visual stimulation (FPVS) with EEG. FPVS-EEG is based on the principle that brain activity synchronizes to a periodically flickering stimulus (Adrian and Matthews 1934). Similar to previous studies (Dzhelyova et al. 2016; Leleu et al. 2018), we applied this principle in an oddball paradigm, where we periodically embedded expressive faces in a stream of neutral faces. The periodic presentation at predefined, yet different, base and oddball frequency rates makes FPVS-EEG a highly objective measure that supports direct quantification of the responses. Furthermore, the rapid presentation enables a fast acquisition of many discrimination responses in a short amount of time, with a high SNR. In addition, FPVS-EEG allows the collection of discriminative responses not only at a group level, but also at an individual level. Individual assessments may help us gain more insight in the heterogeneity within the autism spectrum.

## Present Study Design

We applied FPVS-EEG in boys with and without ASD to quantify and understand the nature of the facial emotion processing difficulties in autism. We implemented fear as the deviant expression between series of neutral faces, because of its potential to elicit large neurophysiological responses (Nuske et al. 2014; Smith 2012). By using neutral faces as forward and backward masks for the fearful faces in a rapidly presented stream (i.e. images are only presented for about 167 ms), the facial emotion processing system is put under tight temporal constraints (Alonso-Prieto et al. 2013; Dzhelyova et al. 2016). This allows us to selectively isolate the sensitivity to the expression.

Based on the literature, we expect a lower neural sensitivity (i.e. reduced EEG responses) for fearful expressions in children with ASD as compared to TD. Detection (i.e. the ability to notice that an emotional content is displayed in a facial expression) of fearful faces can occur without emotion categorization (i.e. the appraisal of which specific expression is shown) (Frank et al. 2018; Sweeny et al. 2013). Therefore, where possible group differences in emotion categorization might be concealed because of floor effects in both groups due to the ongoing development of fear recognition abilities, we expect that FPVS-EEG will reveal possible group differences in the implicit detection of rapidly presented fearful faces. In addition, series of upright as well as inverted faces are presented to assess possible differences in perceptual strategies. Here, we expect to observe more pronounced inversion effects in TD as compared to ASD children. Finally, we investigate whether the detection of a fearful face is modulated by directing the participants’ attention to the eyes versus the mouth of the target face, by placing the fixation cross either on the nasion (i.e. nose bridge) or on the mouth of the face stimuli. This should inform us about the most informative facial cue for fear detection, and whether this most informative cue differs for children with ASD versus TD.

## Methods

### Participants

We recruited 46 8-to-12 year old boys without intellectual disability (FSIQ ≥ 70), comprising 23 TD boys and 23 boys with ASD. Given the higher prevalence of ASD in males (Haney 2016; Loomes et al. 2017) and to avoid confounds due to gender effects on facial emotion processing (McClure, 2000), we only included boys in this study. In addition, given the “own-culture advantage” of emotion processing (Elfenbein and Ambady 2002; Gendron et al. 2014), participants had to be living in Belgium for at least 5 years.

Children with ASD were recruited via the Autism Expertise Centre at the University Hospital and via special need schools. TD participants were recruited via mainstream elementary schools and sport clubs. Four out of the 46 children were left-handed (2 TD), and three children reported colour blindness (1 TD). Because this did not affect their ability to detect the colour changes of the fixation cross, these participants were not excluded. All participants had normal or corrected-to-normal visual acuity. Five participants with ASD had a comorbid diagnosis of ADHD and seven participants of this group took medication to reduce symptoms related to ASD and/or ADHD (methylphenidate, aripiprazole).

Exclusion criteria were the suspicion or presence of a psychiatric, neurological, learning or developmental disorder (other than ASD or comorbid ADHD in ASD participants) in the participant or in a first-degree relative. To be included in the ASD group, the children needed a formal diagnosis of ASD, established by a multidisciplinary team, according to DSM-IV-TR or DSM-5 criteria (American Psychiatric Association 2000, 2014). Furthermore, the Dutch parent version of the Social Responsiveness Scale (SRS; Roeyers et al. 2012) was used to measure ASD traits in all participants. A total *T*-score of 60 was employed as cut-off for inclusion, with all ASD children scoring above 60 and all TD children scoring below 60 to exclude the presence of substantial ASD symptoms.

Both participant groups were group-wise matched on chronological age and IQ. Participant demographics and descriptive statistics are displayed in Table 1.

**Table 1:** Characteristics of the participant groups

Measures	ASD group (*N* = 23) Mean (SD)	TD group (*N* = 23) Mean (SD)	Statistical comparison^a^	*p*
Age (years)	10.5 (1.4)	10.5 (1.4)	*t*(44) = 0.11	0.91
Verbal IQ^b^	107 (11)	112 (11)	*t*(44) = − 1.44	0.16
Performance IQ^b^	104 (15)	108 (10)	*t*(44) = − 1.16	0.25
Full-scale IQ^b^	106 (9)	110 (9)	*t*(44) = − 1.68	0.10
Social Responsiveness Scale Total (*T* score)	85 (12)	42 (6)	*z* = 3.39	0.000***
Social communication and interaction (*T* score)	83 (12)	41 (7)	*z* = 3.39	0.000***
Restricted interests and repetitive behaviour (*T* score)	85 (11)	45 (4)	*z* = 3.39	0.000***

^a^Statistical analyses by means of two-sample *t* test or Kolmogorov–Smirnov Z test (based on assumptions of normality and equal variances)

^b^Intelligence was assessed using an abbreviated version (Sattler 2001) of the Wechsler Intelligence Scale for Children, third edition (WISC-III-NL; Wechsler 1992) with subscales Picture Completion, Block Design, Similarities, and Vocabulary. Participants were identical to the sample included in the study of Vettori et al. (2018), with the exception of four boys with ASD and two TD boys

***p < 0.001

The Medical Ethical Committee of the university hospital approved this study. Written informed consent according to the Declaration of Helsinki was gathered from the participants and their parents prior to participation.

### Stimuli

The stimuli comprised a subset of the stimuli used by Dzhelyova et al. (2016). Full front images of a neutral and a fearful expression of four individuals—two males, two females—were selected from the Karolinska Directed Emotional Faces database (AF01, AF15, AM01, AM06, (Lundqvist et al. 1998)). The colored images were set to a size of 210 × 290 pixels, equalizing 4.04° × 5.04° of visual angle at 80 cm viewing distance, and were placed against a gray background (RGB = 128, 128, 128; alpha = 255). The facial stimuli varied randomly in size between 80 and 120% of the original size. Mean pixel luminance and contrast of the faces was equalized during stimulus presentation.

### Design

The design was similar to recent studies with fast periodic oddball paradigms (Dzhelyova et al. 2016; Vettori et al. 2019). The experiment consisted of four conditions—based on the orientation of the faces (upright or inverted) and the position of the fixation cross (nasion or mouth)—all repeated four times, resulting in 16 sequences. At the beginning of each sequence, a blank screen appeared for a variable duration of 2–5 s, followed by 2 s of gradually fading in (0–100%) of the stimuli. The images were presented for 40 s, followed by 2 s of gradually fading out (100–0%). The order of the conditions was counterbalanced, with the sequences randomised within each condition.

**Fig. 1:**
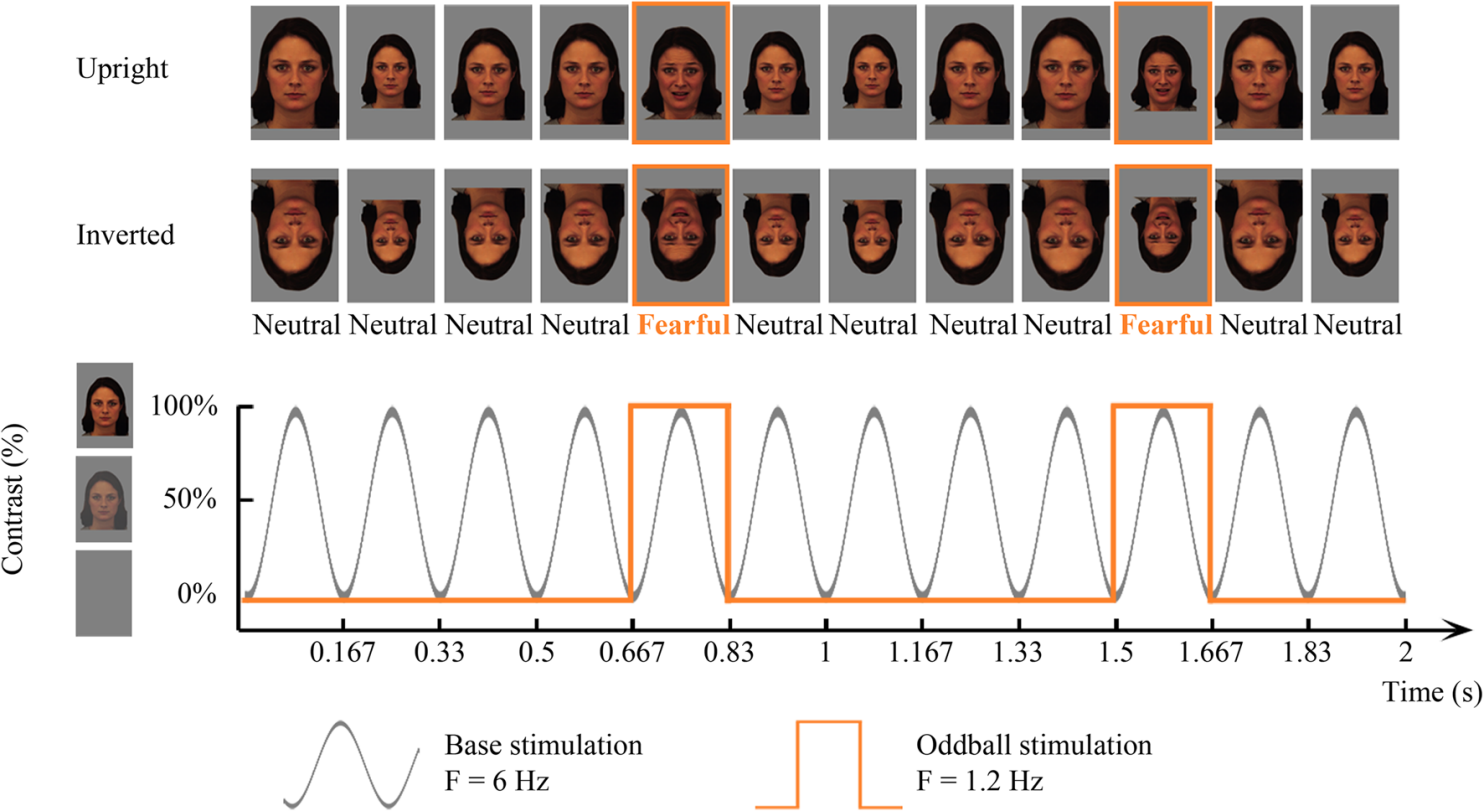
Fast periodic visual stimulation (FPVS) oddball paradigm for the detection of fearful faces, where neutral faces are presented sequentially at a fast 6 Hz base rate, periodically interleaved with a fearful face every fifth image (i.e. 1.2 Hz oddball rate). In separate trials, the faces are presented either upright or inverted and with the fixation cross on the nasion or on the mouth (Dzhelyova et al. 2016)

Stimuli of neutral faces (e.g. individual A) were displayed at a base rate of 6 Hz, periodically interleaved with a fearful oddball stimulus of the same individual every fifth image [6 Hz/5 = 1.2 Hz oddball rate; based on previous research (Alonso-Prieto et al. 2013; Dzhelyova and Rossion 2014a; Liu-Shuang et al. 2014)], generating the following sequence A_neutral_A_neutral_A_neutral_A_neutral_**A**_**fearful**_A_neutral_A_neutral_A_neutral_A_neutral_**A**_**fearful**_ (see Fig. 1 and the Movie in Online Resource 1). A custom application software written in Java was used to present images through sinusoidal contrast modulation (0–100%) (see also Fig. 1).

### Procedure

Participants were seated in a dimly lit room in front of a LCD 24-in. computer screen, which was placed at eye level. To guarantee attentiveness of the participants, an orthogonal task was implemented. A fixation cross, presented either on the nasion of the face or on the mouth, briefly (300 ms) changed color from black to red 10 times within every sequence. The participants had to respond as soon and accurately as possible when noticing the color changes of the fixation cross.

#### EEG Acquisition

We recorded EEG activity using a BIOSEMI Active-Two amplifier system with 64 Ag/AgCl electrodes and two additional electrodes as reference and ground electrodes (Common Mode Sense active electrode and Driven Right Leg passive electrode). We recorded vertical eye movements by positioning one electrode above and one below the right eye; additionally, one electrode was placed at the corner of both eyes to record horizontal eye movements. We recorded EEG and electrooculogram at 512 Hz.

#### EEG Analysis

##### Preprocessing

We processed all EEG data using Letswave 6 (http://www.nocions.org/letswave/) in Matlab R2017b (The Mathworks, Inc.). We cropped the continuously recorded EEG data into segments of 45 s (2 s before and 3 s after each sequence), bandpass filtered it at 0.1–100 Hz using a fourth-order Butterworth filter, and resampled the data to 256 Hz. We applied independent component analysis via the runica algorithm (Bell and Sejnowski 1995; Makeig et al. 1995) to remove blink artefacts for two TD participants who blinked on average more than 2SD above the mean (average number of blinks across participants = 0.19, SD = 0.22). We re-estimated noisy or artifact-ridden channels through linear interpolation of the three spatially nearest, neighboring electrodes. All data segments were re-referenced to a common average reference.

##### Frequency Domain Analysis

The preprocessed data segments were cropped to contain an integer number of 1.2 Hz cycles starting immediately after the fade-in until approximately 39.2 s (47 cycles). Data were then averaged in the time domain, for each participant individually and per condition. A fast fourier transformation (FFT) was applied to these averaged segments, yielding a spectrum ranging from 0 to 127.96 Hz with a spectral resolution of 0.025 (= 1/40 s).

The recorded EEG contains signals at frequencies that are integer multiples (harmonics) of the 6 Hz base stimulation frequency and the 1.2 Hz oddball frequency. To measure the discrimination response to fearful faces, only the amplitude at the frequencies corresponding to the oddball frequency and its harmonics (i.e. n*F/5 = 2.4 Hz, 3.6 Hz, 4.8 Hz, etc.) is considered (Dzhelyova et al. 2016). We used two measures to describe this fear discrimination response: SNR and baseline-corrected amplitudes. SNR is expressed as the amplitude value of a specific frequency bin divided by the average amplitude of the 20 surrounding frequency bins, whereas the baseline-corrected amplitude is calculated by subtracting the average amplitude level of the 20 surrounding bins from the amplitude of the frequency bin of interest (Liu-Shuang et al. 2014). We used SNR spectra for visualization, because the responses at high frequency ranges may be of small amplitude, but with a high SNR. Baseline-correction expresses responses in amplitudes (µV) that can be summed across significant harmonics to quantify an overall base and oddball response (Dzhelyova and Rossion 2014b; Retter and Rossion 2016).

To define the number of harmonics of the base and oddball frequencies to include in the analyses, for each condition we assessed the significance of the responses at different harmonics by calculating Z-scores (Liu-Shuang et al. 2014) on the FFT grand-averaged data across all electrodes and across electrodes in the relevant regions of interest (ROIs; cf. infra). We considered harmonics significant and relevant to include as long as the Z-score for two consecutive harmonics was above 1.64 (*p* < 0.05, one-tailed) across both groups and across all conditions (Retter and Rossion 2016). Following this principle, we quantified the oddball response as the sum of the responses of seven harmonics (i.e. 7F/5 = 8.4 Hz), without the harmonics corresponding to the base rate frequency (F = 6 Hz). The base frequency response was quantified as the summed responses of the base rate and its following two harmonics (2F and 3F = 12 Hz and 18 Hz, respectively).

In addition, analyses were performed at the individual subject level by calculating individual Z-scores for each of the relevant ROIs. We averaged the raw FFT spectrum per ROI and cropped it into segments centered at the oddball frequency and its harmonics, surrounded by 20 neighboring bins on each side that represent the noise level (Dzhelyova et al. 2016; Vettori et al. 2019). These spectra were summed across the significant harmonics and then transformed into a Z-score (see above).

##### Brain Topographical Analysis and Determination of ROIs

Based on visual inspection of the topographical maps and in accordance with the identification of the left and right occipito-temporal region as most responsive for socially relevant oddball stimuli, and the medial occipital region as most responsive for base rate stimulation (Dzhelyova et al. 2016; Vettori et al. 2019), we defined the following ROIs: (1) left and right occipito-temporal (LOT and ROT) ROIs by averaging for each hemisphere the four channels with the highest summed baseline-corrected oddball response averaged across all conditions (i.e. channels P7, P9, PO7 and O1 for LOT, and P8, P10, PO8 and O2 for ROT), (2) medial occipital ROI (MO) by averaging the two channels with the largest common response at 6 Hz (i.e. channels Iz and Oz).

#### Behavioral Facial Expression Measures

Two computerized behavioral facial expression processing tasks were administered.

The Emotion Recognition Task (Kessels et al. 2014; Montagne et al. 2007) investigates the explicit recognition of six dynamic basic facial expressions. Similar to the study of Evers et al. (2015), we applied two levels of emotion intensity: 50% and 100%. Children observe short video clips of a dynamic face in front view (4 clips per emotion), and have to select the corresponding emotion from the six written labels displayed left on the screen. Prior to task administration, participants were asked to provide an example situation for each emotion to ensure that they understood the emotion labels.

In the Emotion-matching task (Palermo et al. 2013) participants have to detect a target face showing a different facial emotion compared to two distractor faces both showing the same expression. The same six emotions as in the Emotion Recognition Task are involved. Here, we used the shorter 65-item version of the task, preceded by four practice trials (for specifics, see Palermo et al. 2013).

#### Statistical Analysis

For the statistical group-level analyses of the baseline-corrected amplitudes, we applied a linear mixed-model ANOVA (function ‘lmer’ (package ‘lme4’) in R (Bates et al. 2015)), fitted with restricted maximum likelihood. Separate models were fitted with either the base or the oddball rate response as the dependent variable. Fixation (eyes vs. mouth), orientation (upright vs. inverted faces) and ROI (LOT, ROT, MO) were added as fixed within-subject factors, and group (ASD vs. TD) as a fixed between-subject factor. To account for the repeated testing, we included a random intercept per participant. Degrees of freedom were calculated using the Kenward–Roger method. Planned posthoc contrasts were tested for significance using a Bonferroni correction for multiple comparisons, by multiplying the p-values by the number of comparisons.

In addition to the group-level analyses, we also evaluated the significance of the fear detection response for each individual participant based on their z-scores. Responses were considered significant if the z-score in one of the three ROIs exceeded 1.64 (i.e. *p* < 0.05; one-tailed: signal > noise).

Subsequently, we applied a linear discriminant analysis (LDA) on the EEG data to classify individuals as either belonging to the ASD or TD group. We carried out a variable selection (‘gamboost’ function in R (Buehlmann et al. 2018)) to identify the most informative predictors, resulting in 12 input vectors for the LDA model—i.e. the first four oddball harmonics for each of the three ROIs. We expect them to be highly correlated, however, these between-predictor correlations are handled by the LDA (Kuhn and Johnson 2013). Before performing the LDA classification, assumptions were checked. A Henze-Zirklers test (α = 0.05) with supplementary Mardia’s skewness and kurtosis measures showed a multivariate normal distribution of the variables. A Box’s M-test (α = 0.05) revealed equal covariance matrices for both groups. In addition, we assessed the competence of the classification model to address the issues of small sample sizes and possible over-fitting by carrying out permutation tests (Noirhomme et al. 2014).

For the behavioral data of the orthogonal task and the Emotion-matching task, the assumptions of normality and homoscedasticity were checked using a Shapiro–Wilk and Levene’s test, respectively. For normal distributions, an independent-samples *T* test was applied, otherwise, we performed a Mann–Whitney U test. When the assumption of homogeneity of variances was violated, degrees of freedom were corrected using the Welch-Sattertwaite method. For the Emotion Recognition Task, we applied a linear mixed-model ANOVA, with intensity level (50% vs. 100%) and expression (anger, fear, happiness, sadness, disgust, surprise) as fixed within-subject factors and group as between-subject factor. Again, we included a random intercept per participant.

All assumptions in terms of linearity, normality and constance of variance of residuals were verified and met for all linear mixed-model ANOVAs.

Due to equipment failure, data on the Emotion Recognition Task were missing for one TD participant. In addition, data of the Emotion-matching task were discarded for one TD participant because he did not follow the instructions and randomly pressed the buttons.

All analyses have been performed with and without inclusion of colorblind children, ASD children with comorbidities, and ASD children who take medication. As their inclusion/exclusion did not affect any results, we only report results with all participants included.

## Results

### General Visual Base Rate Responses

**Fig. 2:**
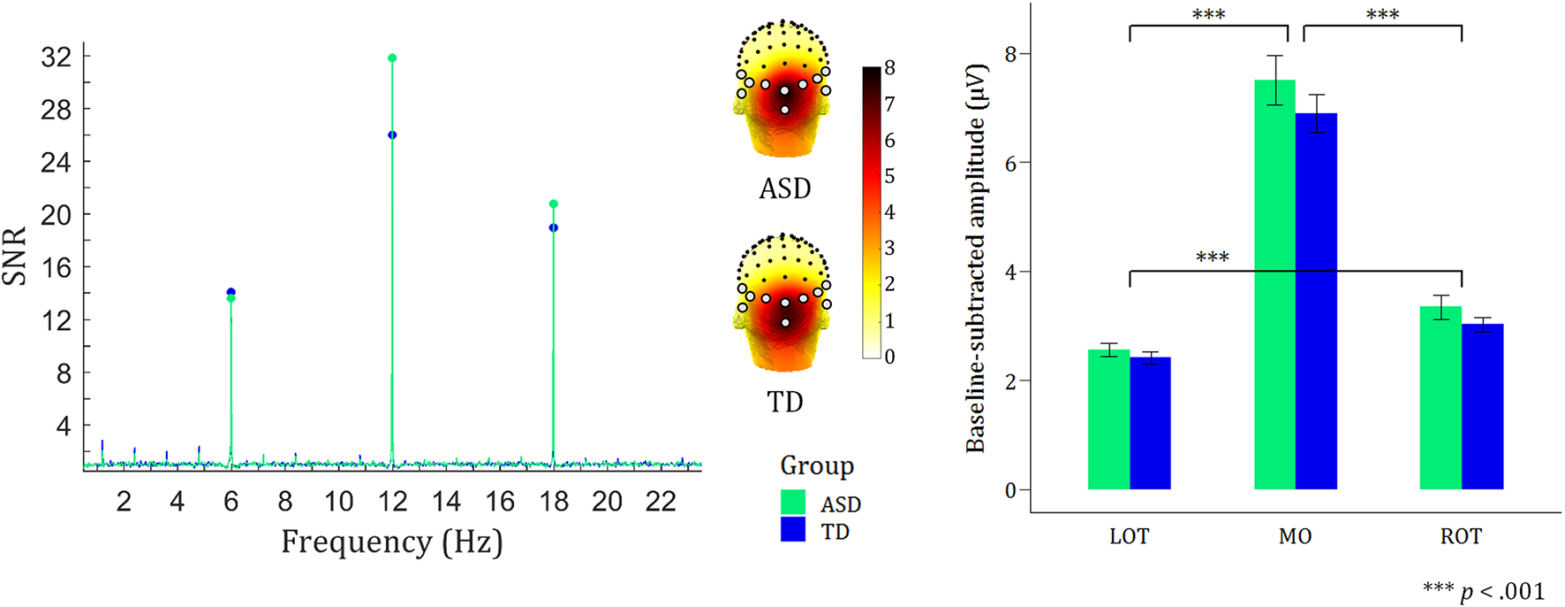
Similar general visual responses to faces in ASD and TDs. *Left* SNR spectrum over the averaged electrodes of the MO region, with clear peaks at the base frequency (6 Hz) and its two subsequent harmonics (12 Hz and 18 Hz). *Middle* Scalp distribution of the general visual base rate responses. The four most leftward and four most rightward open circles on the topographical map constitute LOT and ROT, respectively. The two central open circles constitute MO. *Right* The summed baseline-subtracted amplitudes across the three harmonics of the base rate for each of the three ROIs [medial-occipital (MO) and left and right occipito-temporal (LOT and ROT) regions]. Error bars indicate standard errors of the mean. The main effect of ROI is indicated on the bar graphs, with MO > LOT & ROT, and ROT > LOT

Clear brain responses were visible at the 6 Hz base rate and harmonics, reflecting the general visual response to the faces (Fig. 2). The response was distributed over medial occipital sites. The linear mixed-model ANOVA revealed a highly significant main effect of ROI (F(2,498)= 441.26, *p* = < 0.001), with planned contrasts indicating highest responses in the MO region and lowest responses in the LOT region (M_LOT_ = 2.49 < M_ROT_ = 3.19 < M_MO_ = 7.21; t(498)_LOT–MO_ = − 27.52, t(498)_LOT–ROT_ = − 4.07, t(498)_ROT–MO_ = − 23.45, all *p*_*Bonferroni*_ < 0.001). There were no other significant main and/or interaction effects, suggesting similar synchronization to the flickering stimuli in the two participant groups (all *p* > 0.15).

### Fear Discrimination Responses

**Fig. 3:**
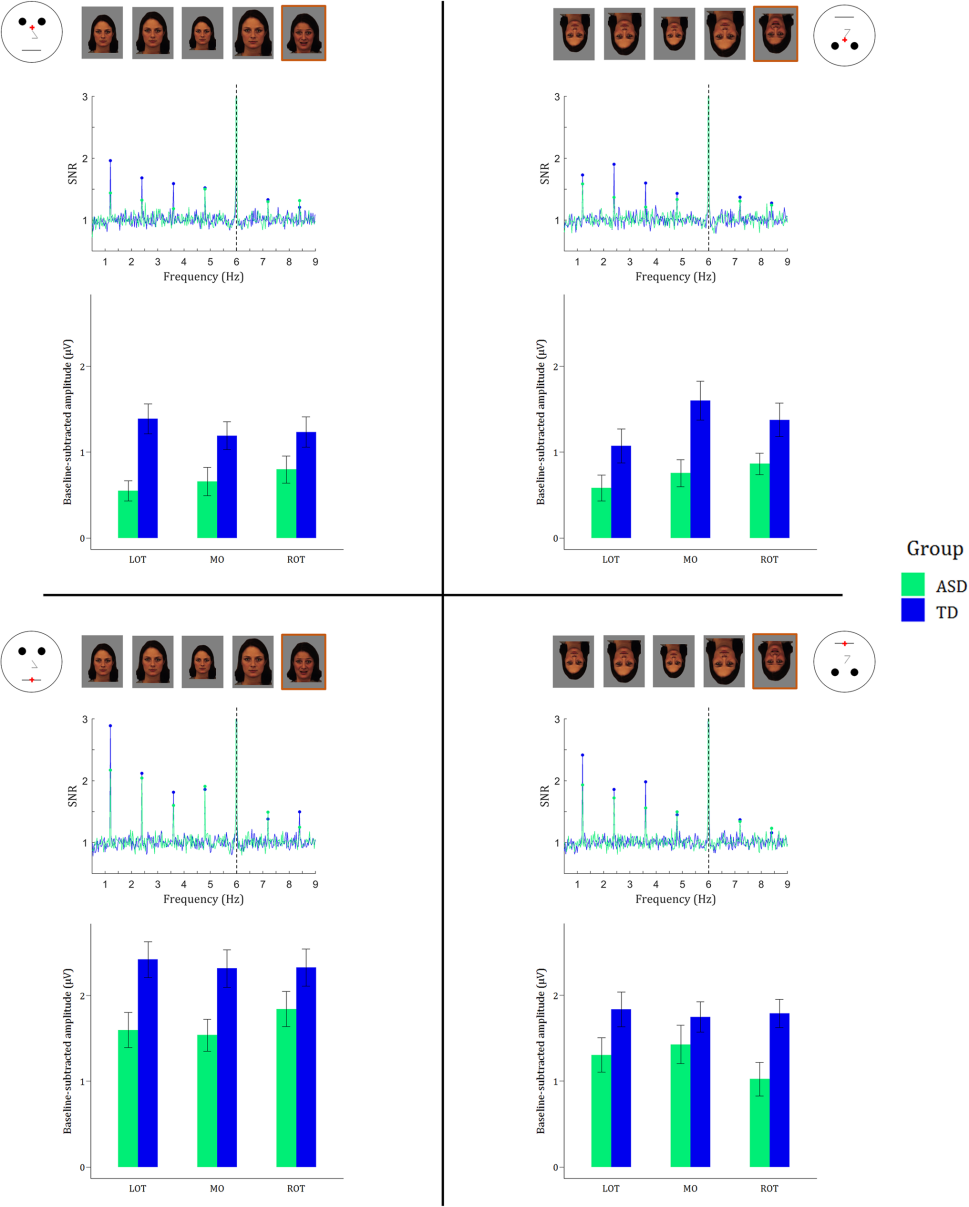
Oddball responses for each experimental condition (based on the orientation of the face and the position of the fixation cross; eye fixation on the top, mouth fixation on the bottom) visualized via two measures: (1) SNR spectra averaged across the three ROIs, and (2) summed baseline-subtracted amplitudes for the seven first oddball harmonics (excluding 6 Hz; i.e. the dashed line) shown in bar graphs. Error bars reflect standard errors of the mean

Figure 3 visualizes clear fear discrimination responses in the four experimental conditions at the oddball frequency and its harmonics.

**Fig. 4:**
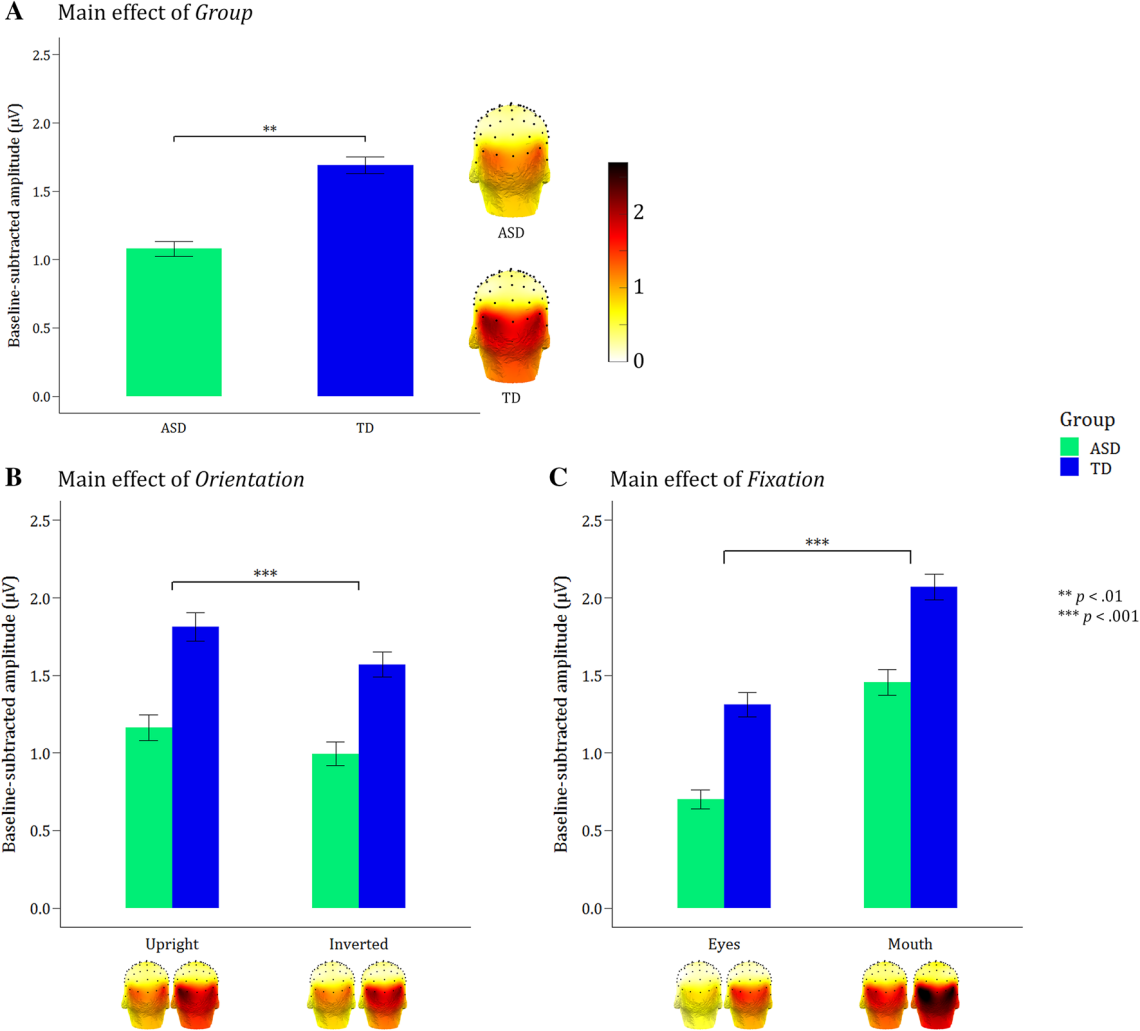
Main effects of group, orientation and fixation. Mean fear discrimination responses (averaged across all three ROIs) of both participant groups in all experimental conditions, visualized via scalp topographies and bar graphs of the summed baseline-subtracted amplitudes for the included oddball harmonics (until 8.4 Hz, excluding the 6 Hz harmonic). Error bars are standard errors of the mean. **a** The main effect of *Group* shows overall higher responses to fearful faces in the TD group compared to the ASD group. These significantly higher responses of the TD group remain visible in all conditions. **b** The main effect of *Orientation* demonstrates a clear inversion effect, with significantly higher fear discrimination responses to upright faces compared to inverted faces. **c** The main effect of *Fixation* reveals significantly higher responses when the fixation cross is placed on the mouth, compared to the eye region

Most importantly, the linear mixed-model ANOVA of the fear detection responses showed a highly significant main effect of group, with higher responses in the TD group (M_TD_ = 1.69) versus the ASD group (M_ASD_ = 1.08, *F*(1,44) = 12.17, *p* = 0.001; Fig. 4a). Additionally, the main effect for orientation of the presented faces (*F*(1,498) = 11.52, *p* < 0.001) indicated higher fear discrimination responses for upright versus inverted faces (M_inverted_ = 1.28 < M_upright_ = 1.49; Fig. 4b). The main effect of fixation (*F*(1,498) = 155.51, *p* < 0.001) demonstrated much higher discrimination responses when the fixation cross is placed on the mouth versus the eyes (M_eyes_ = 1.01 < M_mouth_ = 1.76; Fig. 4c). The absence of interactions with Group (all *p *> 0.56) indicated that all these effects were equally present in the TD and the ASD group. The linear mixed-model ANOVA yielded no main effect of ROI (*p* > 0.63).

Thus, the group analysis revealed large and significant quantitative differences in the amplitude of the fear discrimination response between TD and ASD. Yet, it is also important to investigate to what extent reliable fear discrimination responses can be recorded at the individual subject level. Statistical analysis of the individual subject data confirmed that all subjects but one boy with ASD (45/46) displayed a significant discrimination response for the most robust condition with upright faces and fixation cross on the mouth (z > 1.64, *p* < 0.05). See Table 2 for the results in all conditions.

**Table 2:** Number of individuals displaying significant individual fear discrimination responses

	ASD (*N* = 23)	TD (*N* = 23)
Upright + mouth	22	23
Upright + eyes	16	23
Inverted + mouth	20	23
Inverted + eyes	17	21

Based on statistical analysis of the individual subject data. Fear discrimination responses were considered significant with z-scores > 1.64 (*p* < 0.05)

**Fig. 5:**
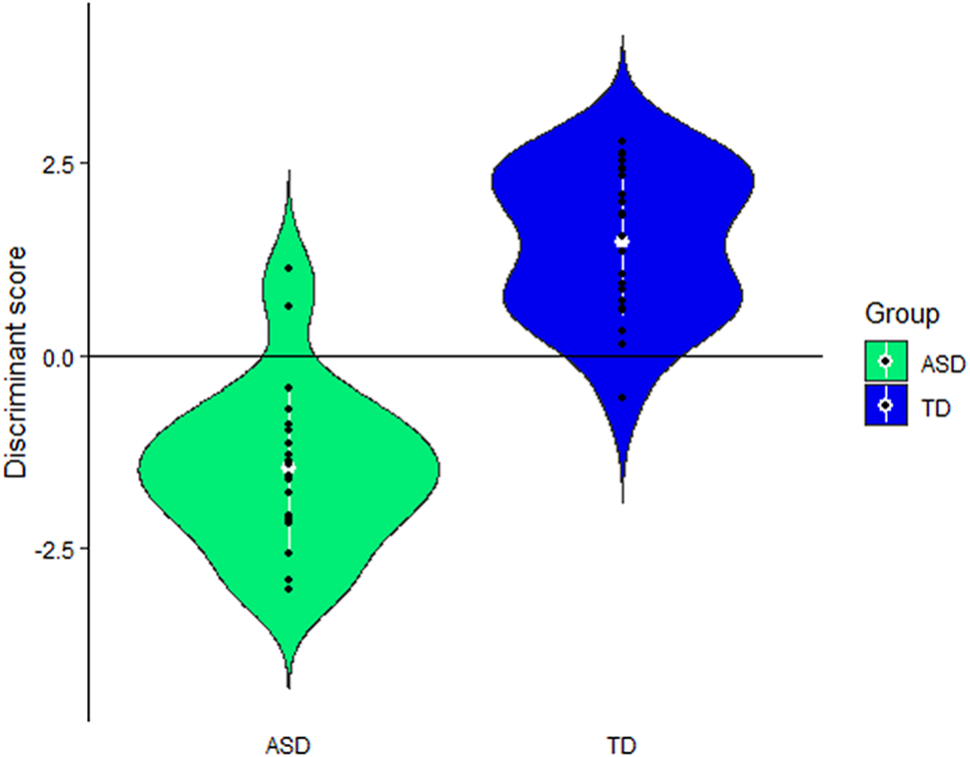
Violin plot of the LDA classification. The horizontal line represents the decision boundary of the LDA classifier and illustrates the differentiation between the two groups. When fitted to the full dataset, the LDA classifies 21 out of 23 participants with ASD and 22 out of 23 TD participants correctly. In white: mean ± 1 SD

Thus far, a reliable biomarker to distinguish people with and without ASD has not yet been established (Raznahan et al. 2009). To qualify as biomarker, objective quantifications of biological and functional processes are needed at the individual level (Mcpartland 2016; McPartland 2017), rather than mere statistical group differences. To evaluate the potential of our fear detection paradigm as a sensitive and objective marker of clinical status, we analyzed how well these responses can predict group membership of our participants. To understand how well the LDA classification generalizes, we relied on a leave-one-out cross-validation, which estimated an overall accuracy of 83% of the LDA model to predict group membership. More specifically, the sensitivity (i.e. correctly classifying individuals with ASD in the ASD group) and specificity (i.e. correctly classifying TD boys in the TD group) were estimated at 78% and 87%, respectively. The linear differentiation between both groups based on the full dataset is shown in Fig. 5. Statistical assessment of the competence of the classification model demonstrated a likelihood of obtaining the observed accuracy by chance of *p* < 0.0001 for 10,000 permutations and additional inclusion of the neural responses of either the 7.2 Hz oddball harmonic or both the 7.2 Hz and 8.4 Hz oddball harmonics.

### Behavioral Measures: Orthogonal Task and Explicit Facial Emotion Processing

Results from the Mann–Whitney U test demonstrated equal accuracy (M_ASD_ = 90%, SD = 12; M_TD_ = 93%, SD = 6.8; W = 215, *p* = 0.54) and reaction times (M_ASD_ = 0.53 s, M_TD_ = 0.48 s, W = 296, *p* = 0.21) for both groups on the fixation cross color change detection task, suggesting a similar level of attention throughout the EEG experiment.

For both explicit emotion processing computer tasks, all ASD and TD participants performed above chance level. A mixed-model ANOVA on the accuracy data of the Emotion Recognition Task showed that full-blown expressions were labelled more accurately compared to expressions presented at 50% intensity (*F*(1,478) = 5.59, *p* = 0.019). A main effect of emotion (*F*(5,478) = 76.32, *p* < 0.001) revealed that happy and angry faces were most often labelled correctly, whereas fearful and sad faces were the most difficult to label correctly. The main effect of group and the interaction effects were not significant (all *p* > 0.40). To ensure that results were not driven by differential response biases, we calculated how often specific emotion labels were chosen by each individual. Since we did not find group differences in response bias (see Appendix Table 3), there was no need to repeat the analysis with corrected performances.

Whereas both participant groups showed equal performance in terms of emotion labelling, a significant group difference was found for the matching of expressive faces, with the TD group outperforming the ASD group (M_ASD_ = 63%, SD = 11.0; M_TD_ = 69%, SD = 6.8; t(37.01) = − 2.29, *p* = 0.028). No differences were found in reaction times (M_ASD_ = 4.27 s, M_TD_ = 4.24 s, t(41.78) = 0.08, *p* = 0.94).

## Discussion

With FPVS-EEG, we evaluated the implicit neural sensitivity of school-aged boys with and without ASD to detect briefly presented fearful faces among a stream of neutral faces, and we investigated to what extent this sensitivity is influenced by the orientation of the face and by attentional focus to the eye versus mouth region. In addition, we analyzed the performance of both groups on two explicit tasks: an emotion labeling and an emotion matching task.

No group differences were found for the general visual base rate responses, indicating that the brains of children with and without ASD are equally capable of synchronizing with the periodically flickering stimuli, irrespective of the position of the fixation cross or the orientation of the presented faces. However, examination of the responses to changes in expression did reveal differences. We found an overall lower sensitivity to detect fearful faces in boys with ASD as compared to TD boys, regardless whether the faces were presented upright or inverted, or whether attention was oriented towards the eye or the mouth region. As there were no group differences in accuracy and response time of the performances on the orthogonal task, there is no evidence of less attention or motivation of the ASD participants. Analysis of the effects of the experimental conditions showed similar effects in both groups, with higher discrimination responses for upright versus inverted faces, and higher discrimination responses for fixations focused on the mouth versus the eyes. Results of the Emotion Recognition Task showed an equal performance in both groups, with a more accurate performance on the full blown versus half intensity expressions, and with more accurate labelling of happy and angry expressions as compared to sad and fearful expressions. Results on the Emotion-matching task did reveal a group difference, with the TD group outperforming the ASD group.

### Neural Responses Children Versus Adults

Clear responses to brief changes in facial expressions were visible in both participant groups, indicating that 8-to-12-year old boys can detect rapid changes to fearful expressions. Comparison of the brain responses of the TD boys in our sample with brain responses of healthy adults on an identical FPVS paradigm (Dzhelyova et al. 2016) reveals topographical differences for the oddball, but not for the base rate responses. Base rate responses of both children and adults were recorded over the medial occipital sites, spreading out bilaterally to the left and right occipito-temporal regions, with a right hemisphere advantage. The expression-change responses of adults were distributed over occipito-temporal sites, with a right hemisphere advantage (Dzhelyova et al. 2016), whereas the oddball responses of the children in our study did not show this clear lateralization. The relatively larger involvement of MO in fear detection in children as compared to adults may reflect a relatively larger involvement of the primary visual cortex, and thus low-level visual processing (Dzhelyova et al. 2016; Dzhelyova and Rossion 2014a; Liu-Shuang et al. 2014). Indeed, the neural system involved in (expressive) face processing progressively specializes throughout development (Cohen Kadosh and Johnson 2007; Leppänen and Nelson 2009), which is mirrored by a shift in neural activation from a broader medial distribution in childhood to a more focused (bi-)lateral or unilateral distribution in adulthood (de Haan 2011; Dzhelyova et al. 2016; Taylor et al. 2004).

The typical age-related improvement in facial emotion processing (Herba et al. 2006; Herba and Phillips 2004; Luyster et al. 2017) seems to be absent (Gepner 2001; Rump et al. 2009), or at least less pronounced (Trevisan and Birmingham 2016) in individuals ASD. For example, although results are mixed, different latencies and/or amplitudes for the N170 component in ASD, relative to TDs, have been reported from early childhood (Dawson et al. 2004), extending throughout adolescence (Batty et al. 2011; Wang et al. 2004). However, different results when matching participants on verbal or mental age instead of chronological age suggest a developmental delay in specialized facial expression processing in children with ASD (Batty et al. 2011; De Jong et al. 2008), but the neural mechanisms across the developmental trajectory of facial expression processing in ASD remain unclear (Leung 2018). Therefore, from a developmental perspective, applying this paradigm in children, adolescents and adults with ASD could clarify the course of the atypical maturation in individuals with ASD.

### Reduced Neural Sensitivity to Fearful Faces in ASD

In terms of topographical distribution of the selective neural response to fearful faces, there is no difference between the ASD and TD group, suggesting the use of a similar emotional face processing network. However, given the progressive development of emotional face processing capacities in childhood, potential group differences in topography may still appear in adolescence and adulthood.

Turning towards the size of the selective response to the fearful faces, we do observe clear group differences, with lower amplitudes in the ASD sample. Given that adults with ASD display impaired emotion detection (Frank et al. 2018), it is not surprising that a deficit in this ability is already present during childhood. Importantly, the reduced neural sensitivity for detecting fearful faces among a stream of neutral faces is not due to deficits in implicitly detecting oddball categories per se. Indeed, a parallel study on a related group of 8-to-12 year old boys with ASD versus TD matched controls (Vettori et al. 2019) does show intact generic face categorization responses in children with ASD, indicating an equal sensitivity to implicitly detect faces within a stream of non-social images. However, boys with ASD were clearly less sensitive to detect the more subtle socio-communicative cues signaling the appearance of a different facial identity (Vettori et al. 2019). In the present study, we only used fearful faces to investigate facial expression discrimination. Including other emotions as well could elucidate whether facial emotion detection deficits in individuals with ASD are specific for fear, or if results may generalize to other facial expressions.

Previous studies have shown that age (Lozier et al. 2014; Luyster et al. 2017) and intellectual ability (Hileman et al. 2011; Trevisan and Birmingham 2016) might influence emotion processing performance. As our participant groups were closely matched on age and IQ, the observed group difference in neural sensitivity to fearful faces cannot be attributed to these factors. Likewise, neither can the group difference be driven by a reduced focus of attention in the ASD group, given the equal performances of both groups on the orthogonal task. Five participants with ASD had a comorbid ADHD diagnosis, which may influence attention and be associated with emotion recognition deficits (Tye et al. 2014). Yet, exclusion of these participants did not alter the findings in any way, indicating that the observed group difference in oddball responses is strong and not driven by comorbid ADHD.

Another factor that could explain the differences in fear detection is social functioning. Social functioning has been found to be related to emotional face processing on the neural level (Dawson et al. 2004; Yeung et al. 2014). As evaluating this factor was out of the scope of our study, we did not collect early personal data on the social behavior of our participants, other than the SRS, nor did we administer additional behavioral tasks that could have tapped more into the social skills. Yet, future studies could further explore if and how differences in social functioning affect emotion perception.

### Inversion Affects Fear Detection

Face processing, both in terms of identity and expressions, typically involves a holistic/configural approach (Rossion 2013; Tanaka and Farah 1993). Accordingly, performance is typically disrupted by inverting faces and thereby forcing the use of a less efficient and more feature-based approach, i.e. the face inversion effect (Rossion 2008; Tanaka and Simonyi 2016). Previous studies with similar FPVS-EEG paradigms have indeed demonstrated significantly reduced oddball responses for identity (Liu-Shuang et al. 2014; Vettori et al. 2019) and emotion (Dzhelyova et al. 2016) discrimination in TD children and adults, respectively, when faces are presented upside-down compared to upright. Moreover, the study of Vettori et al. (2019) showed a strong inversion effect for facial identity discrimination in TD boys and an absent inversion effect in boys with ASD. These findings were interpreted as evidence for holistic face perception in TD, and a more feature-based face processing strategy in ASD (Vettori et al. 2019). In the current study, we find a significant face inversion effect in both the TD and ASD sample, suggesting that both groups generally apply a holistic facial expression processing approach, additionally supported by an effective feature-based approach. There is evidence that facial expression processing—and in particular fear detection (Bombari et al. 2013)—is more strongly determined by the processing of specific salient facial features instead of the configural relationship between those features (Sweeny et al. 2013). In our study, for instance, the open mouth in the fearful faces might have facilitated fear detection, also in the inverted condition.

### Directing Attention to the Mouth Facilitates Fear Detection

Evidence regarding the role of the eyes versus the mouth in fear recognition is mixed (Beaudry et al. 2014; Eisenbarth and Alpers 2011; Guarnera et al. 2015). In a similar vein, even though reduced eye contact is one of the clinical criteria of ASD (American Psychiatric Association 2014), the empirical evidence that individuals with ASD focus less on the eyes and more on the mouth is not unequivocal (Bal et al. 2010; Black et al. 2017; Guillon et al. 2014; Nuske et al. 2014). Here, we do find higher fear discrimination responses in boys with ASD when their attention is directed towards the mouth instead of the eyes, which suggests that the mouth region is more informative for them than the eye region. However, rather unexpectedly, this was also the case in the TD group. Apparently, for both groups of children, the mouth is a more salient cue to rapidly detect fearful faces than the eyes. It has indeed been suggested that the mouth is the most informative area for expression processing (Blais et al. 2012) and that, when presented opened, it might enhance early automatic attention (Langeslag et al. 2018). Especially the presence of teeth tends to augment neural responses to expressive faces (DaSilva et al. 2016). The occurring contrast of white teeth against a darker mouth opening and lips might draw the attention. Although the images in our study were presented at a very fast rate, these low-level changes of the fearful mouth might elicit larger responses.

### Implicit Versus Explicit Emotion Processing

The contradicting findings on the behavioral face processing tasks align with the generally mixed findings in previous behavioral research (Lacroix et al. 2014; Uljarevic and Hamilton 2013). Contrary to the implicit FPVS-EEG paradigm, explicit tasks allow the use of various verbal, perceptual and cognitive compensatory strategies (Harms et al. 2010), possibly aiding individuals with ASD to compensate for their intrinsic emotion processing deficits (Frank et al. 2018). These compensatory mechanisms, as well as task characteristics, could account for the mixed findings on behavioral discrimination between ASD and TD individuals (Jemel et al. 2006; Lozier et al. 2014; Nuske et al. 2013; Uljarevic and Hamilton 2013), indicating the limited sensitivity of (certain) behavioral measures to pinpoint the socio-communicative impairments of individuals with ASD (Harms et al. 2010).

The (small) group difference found on the matching task might relate to the more feature based approach used by the ASD children to process facial expressions. As the target faces are paired with maximally confusable distractor emotions, involving similar low-level features (Palermo et al. 2013), reliance on the separate facial features instead of the configuration of the facial expressions may hamper accurate emotion matching in the ASD group.

### Future Research

In addition to the behavioral emotion matching or labelling tasks, an additional explicit emotion detection task at the same rapid presentation rate might allow to compare the implicit and explicit emotion discrimination abilities more directly in these samples.

Our results support the sensitivity of the FPVS-EEG approach to rapidly detect and quantify even small responses at an individual level (Dzhelyova et al. 2016; Liu-Shuang et al. 2014; Liu-Shuang et al. 2016). Furthermore, the predefined expression change frequency allows a direct and objective measurement of the discrimination response, without the complexity of post hoc subtraction of the responses (Campanella et al. 2002; Gayle et al. 2012; Stefanics et al. 2012). It also tackles the hurdle of subjectively defining various components and time windows as is done with the standard ERP approach (Kremlácek et al. 2016). Another asset of the FVPS-EEG approach is the fast acquisition of profuse discrimination responses in a short amount of time, because of the rapid stimulus presentation and the high signal-to-noise ratio. Whereas many trials and long recordings are needed in typical ERP studies to obtain reliable responses, we only need four stimulation sequences of 40 s to reliably measure implicit neural discrimination. All these advantages make it a suitable approach for studying populations that are often difficult to include in research, such as infants and people with low-functioning ASD. Furthermore, the promising result of the LDA classification shows the potential of this technique (possibly by combining several paradigms) to serve as a biomarker for ASD. However, to fully understand the potential of FPVS-EEG as a biomarker for socio-communicative deficits, more research is needed in (clinical) samples with a different age and/or IQ.

## Conclusions

Our results indicate that children with ASD are less sensitive to rapidly and implicitly detect fearful faces among a stream of neutral faces, possibly contributing to difficulties with emotion processing. Both children with and without ASD apply a combined holistic and feature-based processing style, and rely mostly on information from the mouth region to detect the fearful faces.

The advantages of FPVS-EEG with its implicit nature, the strength of the effects, and its straightforward application and analysis, pave the way to including populations that are often excluded from studies because of verbal or cognitive constraints.

### Electronic supplementary material

Below is the link to the electronic supplementary material.

Supplementary material 1 (MOV 37506 kb)

## Appendix

See Table 3.

**Table 3:** Response bias ERT

Responses	Mean ASD (SD)	Mean TD (SD)	Mann–Whitney U (W)	*p*
Fear	5.8 (2.9)	5.1 (2.8)	297.5	0.31
Anger	10.2 (2.8)	9.8 (4.0)	288.5	0.42
Happiness	10.7 (2.5)	10.8 (2.5)	251.0	0.97
Sadness	2.8 (1.8)	3.0 (2.2)	247.5	0.91
Surprise	8.1 (2.8)	8.7 (3.0)	211.0	0.34
Disgust	10.4 (5.1)	10.4 (4.0)	230.0	0.61

Based on the assumptions of normality and equal variances, a Mann–Whitney U test to check for possible response bias between the ASD and TD group revealed no differences
